# Assessing disease activity in inflammatory arthritis using optical spectral transmission: a systematic review compared to joint ultrasound, MRI, and clinical activity markers

**DOI:** 10.1186/s41927-026-00635-x

**Published:** 2026-03-18

**Authors:** Ann-Kathrin Druck, Chandana Keshavamurthy, Mohammed Alhaddad, Andreas V. Goules, Andreas Schwarting, Konstantinos Triantafyllias

**Affiliations:** 1 Division of Rheumatology and Clinical Immunology, Department of Internal Medicine I, University Medical Center of the Johannes Gutenberg University Mainz, Langenbeckstr. 1, 55131 Mainz, Germany; 2 Department of Rheumatology, Banner University Medical Center, University of Arizona, Phoenix, 85284 USA; 3 Department of Pathophysiology, School of Medicine, National and Kapodistrian University of Athens, Athens, Greece

**Keywords:** Optical spectral transmission, Handscan, Rheumatoid arthritis, Inflammatory arthritis, Joint ultrasound, Joint inflammation

## Abstract

**Background:**

Early and accurate detection of inflammatory activity is essential in inflammatory arthropathies, as timely treatment can prevent irreversible joint damage. Optical spectral transmission (OST), implemented in the HandScan device, is a novel, non-invasive imaging method that uses red and near-infrared light to detect perfusion changes associated with angiogenesis and hypervascularity in inflamed joints. This review summarizes current evidence on OST, assessing its diagnostic performance and clinical utility relative to musculoskeletal ultrasound, magnetic resonance imaging, and conventional clinical activity indices.

**Methods:**

This review was conducted in accordance with the Preferred Reporting Items for Systematic Reviews and Meta-Analyses (PRISMA) guidelines. Relevant studies were identified through systematic searches of the Web of Science and PubMed databases, with the last search conducted on October 7, 2025, according to predefined inclusion criteria. Publications from 2012 to 2025 were considered eligible. Studies enrolling patients with rheumatic diseases were included, and data were extracted on study population characteristics, methodologies, and main findings.

**Results:**

Out of 77 studies screened, 22 met the inclusion criteria. Cross-sectional studies showed significant correlations between OST and ultrasound findings, with areas under the curve (AUC) reaching as high as 0.85. This correlation was particularly strong in the metacarpophalangeal and proximal interphalangeal joints. Additionally, OST correlated with MRI-detected synovitis and tenosynovitis but not with bone marrow edema. Moderate associations were observed between OST and clinical indicators such as DAS28, swollen joint counts, and C-reactive protein levels. Longitudinal analyses revealed significant associations between changes in OST values and changes in clinical activity indices, specifically ΔDAS28, and ΔSJC. However, it was found that absolute OST scores alone were inadequate for distinguishing between different levels of disease activity. Factors such as sex, body mass index, and structural joint damage were identified as confounding variables that influenced the measurements.

**Conclusion:**

OST is a promising, rapid, and operator-independent technique for quantifying joint inflammation in rheumatoid arthritis. Although further evaluation of its stand-alone diagnostic accuracy is needed, its integration with clinical assessment and imaging may enhance the objectivity and efficiency of disease monitoring. Standardized measurement protocols and patient-adjusted thresholds will be essential for broader clinical implementation.

**Supplementary Information:**

The online version contains supplementary material available at 10.1186/s41927-026-00635-x.

## Introduction

Inflammatory arthritis (IA) comprises a group of rheumatic diseases, including rheumatoid arthritis (RA), psoriatic arthritis (PsA), peripheral spondyloarthritides (SpA), gout and other arthropathies [1]. IA can cause joint pain, structural damage, reduced functionality, and systemic manifestations [2–6]. If not treated promptly and properly, IA can progress, leading to disability and impaired quality of life [7, 8]. Therefore, early initiation of effective therapy is essential to prevent disease progression [1, 9].

Treatment can follow a treat-to-target (T2T) approach, where therapy is adjusted to achieve specific outcomes, like low disease activity or remission [10]. The consistent and effective implementation of these T2T strategies requires frequent and close monitoring of disease activity, typically every one to three months, particularly during the early or active phases of the disease [11]. Traditionally, the Disease Activity Score in 28 joints (DAS 28) has been used to assess clinical activity [12]. This score considers several factors including joint swelling (swollen joint counts, SJC), tenderness (tender joint counts, TJC), acute phase reactants [C-reactive protein (CRP) or erythrocyte sedimentation rate (ESR)] and the patient’s global assessment (PtGA) [12]. While the DAS28 is commonly utilized, it has several significant limitations. Notably, it is dependent on the examiner, making it partially subjective, and it may not adequately capture subclinical disease activity [13, 14].

Furthermore, the DAS28 is specifically intended for evaluating disease activity in patients with RA and has not been validated for use in other inflammatory arthropathies [15]. Complementary imaging modalities such as musculoskeletal ultrasound (US) and magnetic resonance imaging (MRI) are highly sensitive for detecting joint inflammation [16]. However, both techniques can be time-consuming, especially when detailed scoring is performed, and MRI also involves significant costs [17]. Additionally, MRI often requires the use of contrast agents and can frequently be performed unilaterally [18]. Given these limitations, there is an unmet need for new diagnostic tools that allow rapid, cost-effective, objective and easy-to-perform assessment of disease activity [19].

**Fig. 1:**
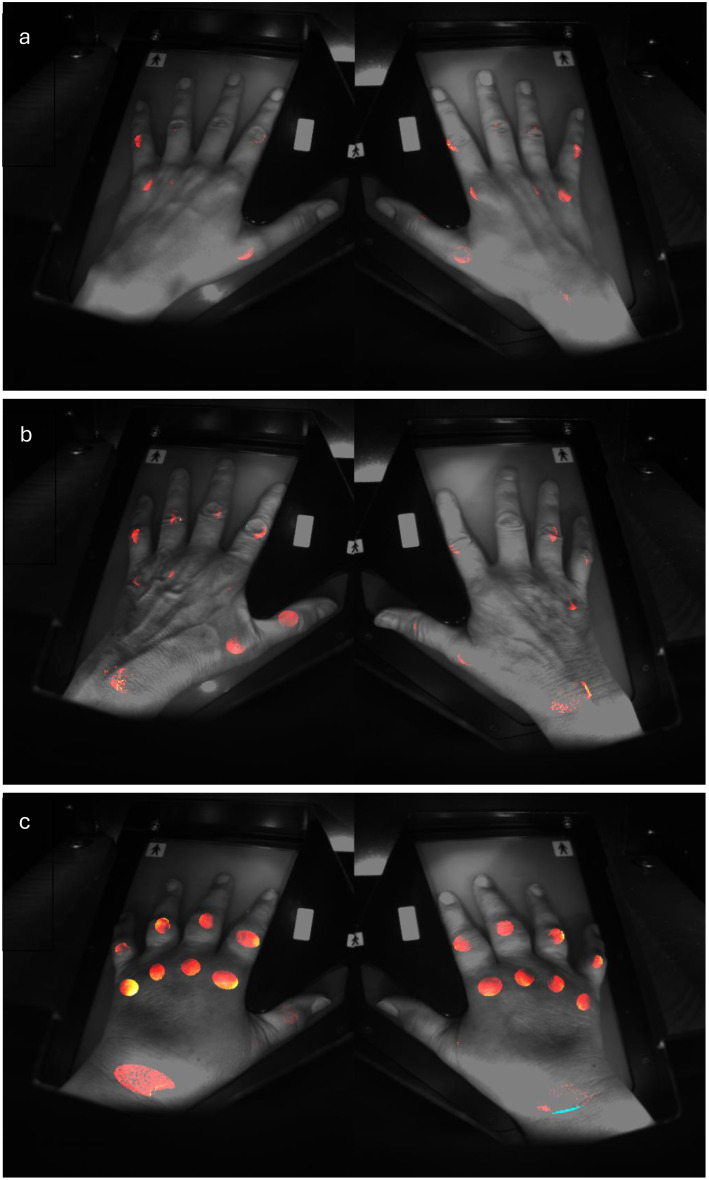
(**a**) Healthy patient; (**b**) Patient with RA in remission; (**c**) Patient with highly active RA

Recently, several new diagnostic technologies have been introduced in rheumatology, including fluorescence optical imaging (FOI) [20–26] and joint-thermography [27–29]. One of these innovations is optical spectral transmission (OST), which is used in the HandScan device [30]. OST is a non-invasive imaging technique that measures the absorption of light through tissue, which is influenced by local blood flow. For this purpose, red and near-infrared laser light wavelengths (typically 660 nm and 808 nm) are utilised [17]. The underlying mechanism is based on the physiological response of inflamed joints: in conditions like RA, joint inflammation leads to increased hypervascularity and angiogenesis in the synovial tissue [31]. Consequently, the blood volume in affected joints increases, resulting in decreased transmission of light through these inflamed areas [32] (Fig. 1).

The OST measurement is non-invasive and painless, taking about 1.5 min to complete, while examining both hands and wrists simultaneously [33]. The HandScan software automatically analyzes acquired data and quantifies inflammation, ensuring operator-independent image interpretation and result generation [14]. Following structured training, the procedure can also be reliably performed by trained healthcare professionals or nursing staff [34] without a specialized rheumatology background.

OST is a promising tool for assessing joint inflammation in inflammatory arthritis; however, several factors have been reported to influence its diagnostic accuracy, including sex, hand size, and the presence of osteoarthritis [33, 35, 36]. Moreover, data regarding OST‘s diagnostic performance are relatively scarce and have not been presented in a collective manner in the scientific literature.

Therefore, this systematic literature review (SLR) aims, for the first time, to thoroughly analyze all published data on OST, assess its diagnostic performance, discuss its limitations, and explore its potential role in current and future rheumatology practice.

## Methods

In this SLR, we implemented a structured search strategy to identify the most relevant publications on the topic. We conducted searches in PubMed and Web of Science using the following combination of keywords and Boolean operators: (“optical spectral transmission” OR “spectral transmission” OR “handscan”) AND (“arthritis” OR “inflammatory arthritis” OR “rheumatoid arthritis” OR “early rheumatoid arthritis” OR “osteoarthritis” OR “psoriatic arthritis” OR “joint inflammation”). This search resulted in 77 studies.

**Fig. 2:**
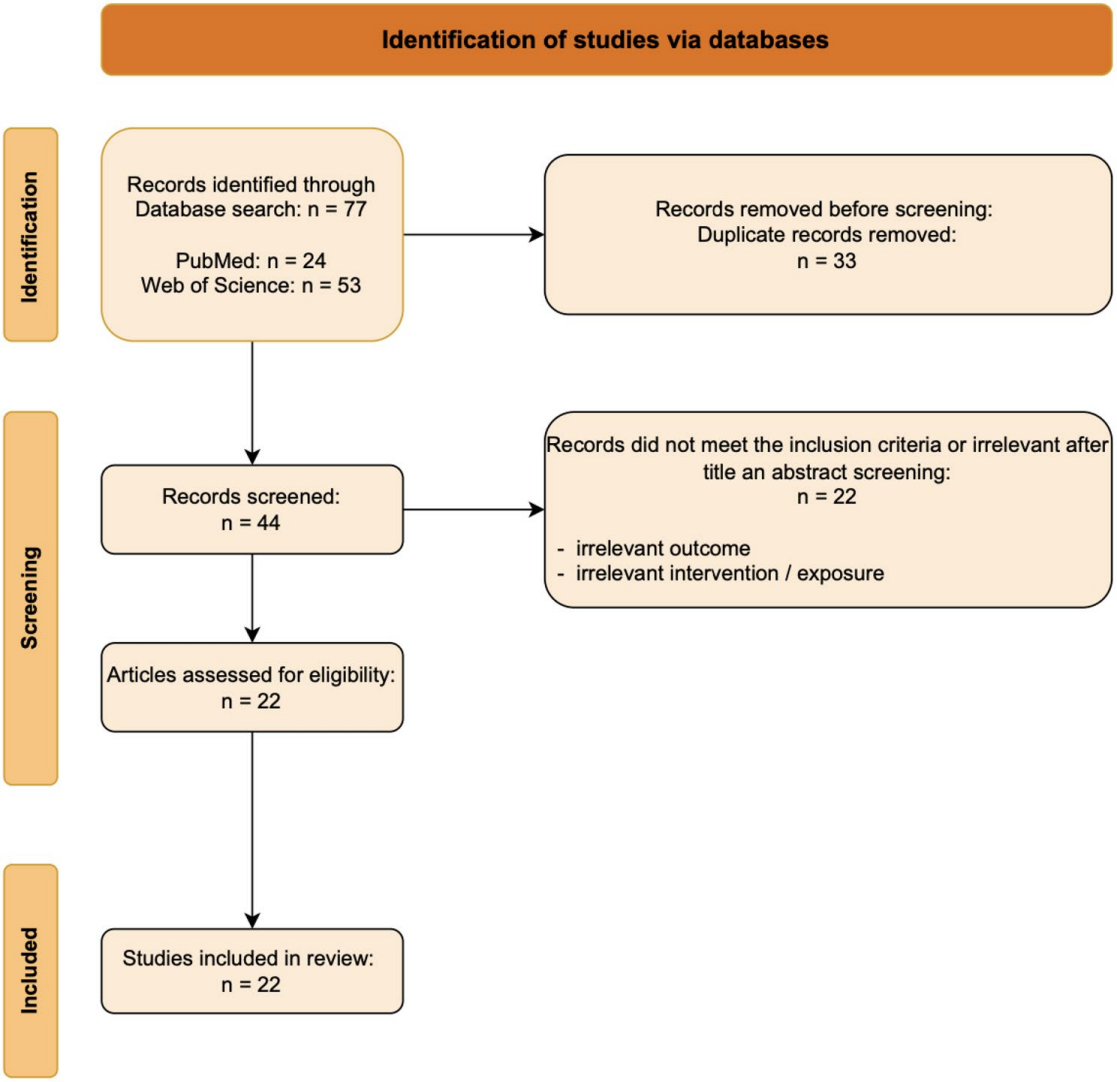
PRISMA 2020 Flow diagram for new systematic reviews which included searches of databases and registers only [37]

Following the PICO framework, we defined the population (P) as patients with rheumatoid arthritis (RA), osteoarthritis (OA), gout, inflammatory arthralgia, or psoriatic arthritis (PsA). The intervention (I) involved OST, while the comparison (C) included assessments through other disease activity measures, such as joint ultrasound, MRI, or DAS28. The outcome was measured using OST scores. We excluded studies published over 15 years ago, and irrelevant and duplicate records were removed manually. In total, 22 studies met the inclusion criteria and were included in this review (Table 1). The detailed selection process is illustrated in the corresponding flow chart (Fig. 2). Screening and data extraction have been performed by two different reviewers (A-K. D. and K.T.). In this SLR, no prospective protocol registration was performed.

## Functional principles of OST

**Fig. 3:**
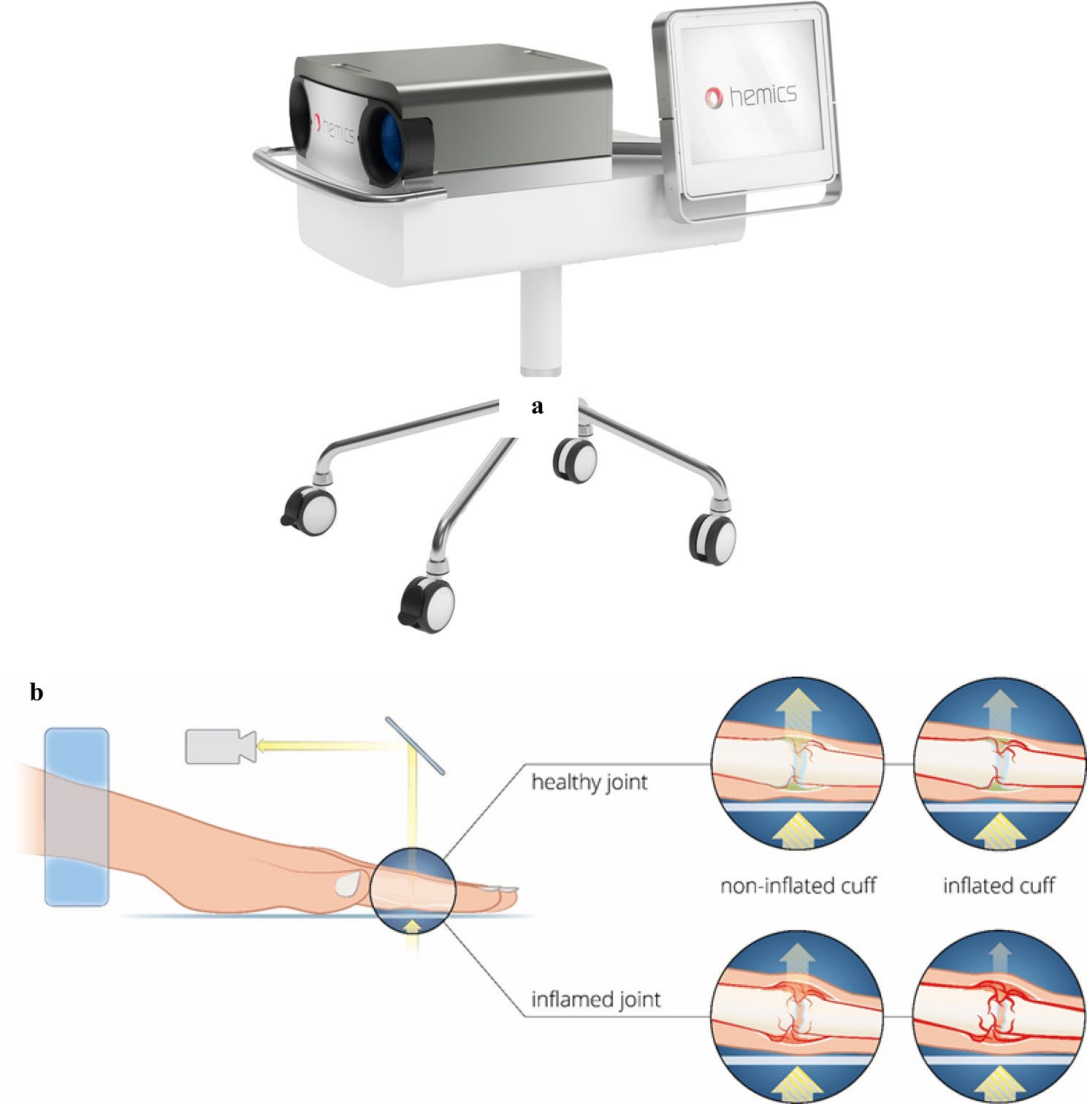
(**a**) Hemics HandScan device; (**b**) The HandScan technology; [42]. Image © Hemics/Demcon, reproduced with permission

The HandScan device consists of a lighting and imaging unit, a hand rest, and a compressed air system [17, 36] (Fig. 3). It functions by quantifying inflammation-related hemodynamic responses to a controlled stimulus [38, 39]. During the procedure, patients places their hands on a hand rest with the sliding cover open. The cover is then closed to initiate the measurement [40]. At first, the pressure cuffs inflate slightly to eliminate any gaps around the arms [40]. Patient’s hands are then illuminated with red and near-infrared light, and the transmitted light is captured by a camera positioned on the opposite side [36]. The integrated camera captures the light transmitted through the hands, which is subsequently analyzed by automated software [41]. The first phase measures baseline transmission and lasts about 10 s, while the second phase takes an additional 60 s [39]. Over the subsequent 60 s, the cuff pressure is gradually increased to 55 mmHg (7.3 kPa) [36]. This occludes venous blood flow, while arterial blood flow can continue to flow unimpeded [30]. This is necessary because it leads to what is known as “bloodpooling,” whereby blood accumulates in the examined joint areas [14]. Interestingly, inflamed joints tend to accumulate more blood and do so more quickly (14.6 s) than non-inflamed joints (21.6 s) [30]. Blood strongly absorbs red and near-infrared light, with blood pooling resulting in a measurable decrease in transmitted light [32, 39] (Fig. 3). The third phase, lasting approximately 30 s, measures the resolution of blood congestion [14].

**Fig. 4:**
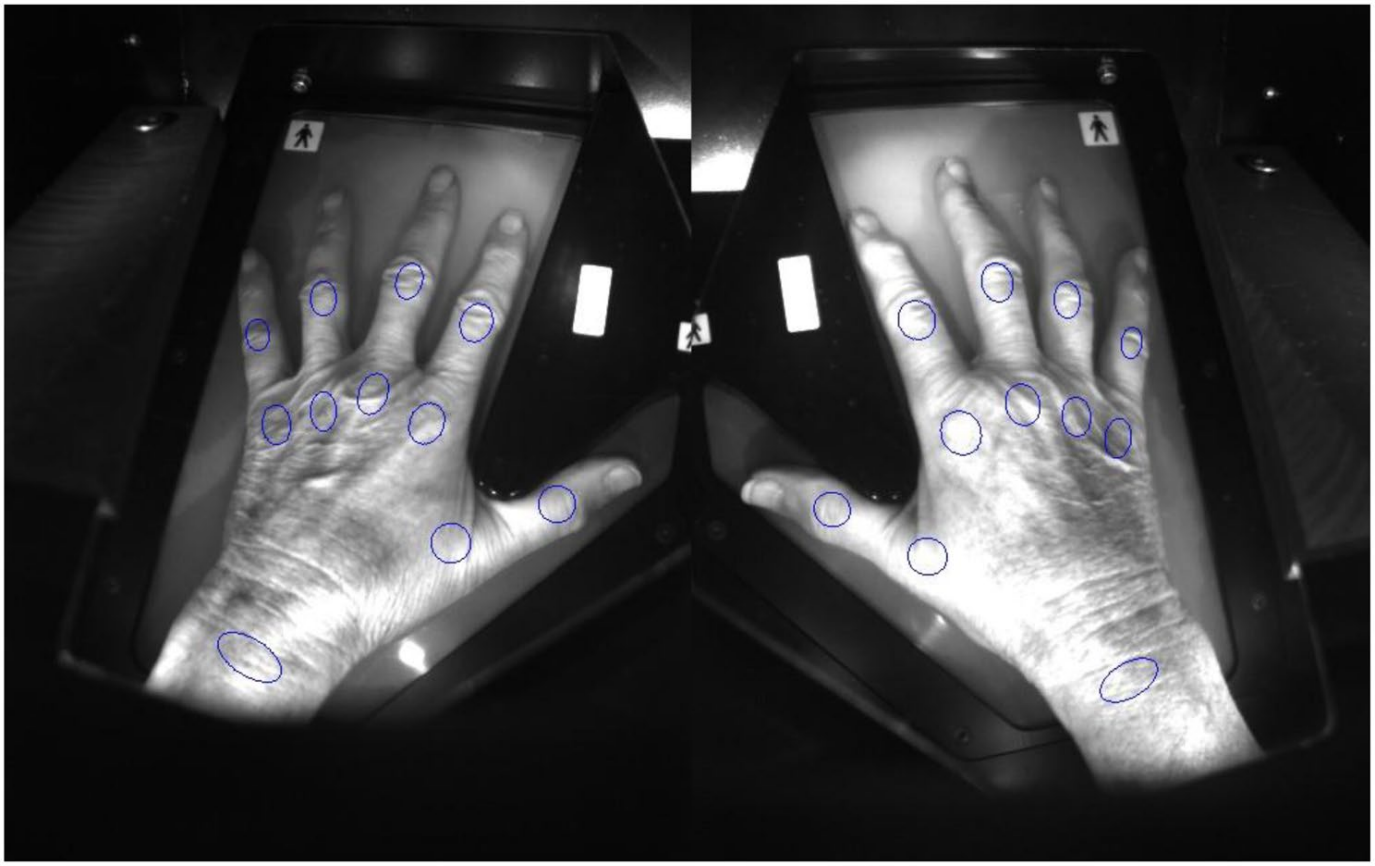
Placement of ROIs in the hand and finger joints of both hands

Due to the vascular changes associated with inflammation, the speed and extent of blood accumulation is different for inflamed and healthy tissue [30, 34]. Overall, a complete measurement takes around 100 s [14]. The device provides an OST index for each of the 22 joints examined -bilateral metacarpophalangeal (MCP) joints, proximal interphalangeal (PIP) joints, and wrists -as well as a total optical score (TOS) for the patient. The resulting TOS is directly inversely proportional to the transmitted light, and since the absorption of light is measured, a higher OST index also indicates a higher level of inflammation [36, 40]. The TOS is calculated as the average of individual joint scores multiplied by 22, ranging from 0 to 66, with 66 representing the highest possible score [17, 32, 33]. Regions of interest (ROIs) are critical for accurate measurement, allowing the user to precisely position measurement points over the joints (Fig. 4). ROIs are automatically generated for each measurement and must be reviewed and, if necessary, adjusted by the user before starting the scan [17, 34, 39].

## Results

The existing literature is divided into two main areas: Cross-sectional studies, which analyse the diagnostic performance and related factors of OST at a specific time point, and longitudinal studies, which evaluate the OST’s ability to track changes in disease activity over time.

## Cross-sectional studies: diagnostic performance, correlations and influencing factors of OST

The included studies investigate how OST values relate to indicators of disease activity and analyze patient’s related factors that may affect these measurements. This analysis is often conducted in comparison to established diagnostic methods, such as clinical examinations, joint ultrasound, or MRI.

### Diagnostic performance of OST compared to US

Joint-US is a well-established tool used for assessing joint inflammation [32]. It is a more sensitive tool for detecting synovitis than clinical examination [43]. In rheumatology practice, both grayscale ultrasound (GSUS) and power Doppler ultrasound (PDUS) are frequently used to evaluate arthritic joints, each providing complementary information [44, 45]. Gray-scale ultrasound (GSUS) is utilised to evaluate morphological and structural changes in joints, allowing for the visualisation of features such as synovial thickening and joint effusion [17]. PDUS evaluates blood flow activity and visualizes the vascularity within the synovial tissue, which increases in inflamed joints and can therefore be quantified [46]. Both GSUS and PDUS findings can be graded using a semi-quantitative scale ranging from 0 to 3, as recommended by Outcome Measures in Rheumatoid Arthritis Clinical Trials (OMERACT) [47, 48].

Six cross-sectional studies have utilised joint ultrasound as the reference standard for OST in patients with inflammatory arthritis [17, 31, 32, 35, 41, 49]. Data on osteoarthritis patients are provided in the supplementary material S1 [38]. The first of these studies, conducted by Krabbe et al., evaluated 62 patients who met the 2010 European League Against Rheumatism (EULAR) and American College of Rheumatology (ACR) criteria for rheumatoid arthritis (RA) [31]. None of the patients had relevant deformities of the hands or wrists, and none had undergone surgery within the previous three months. For each patient, data were collected cross-sectionally using OST, color Doppler ultrasonography (CDUS), and clinical examination. The results were then compared to assess the sensitivity and specificity of OST. When analyzing all joints (wrists, MCP, and PIP joints), OST demonstrated a sensitivity of 54% and a specificity of 79% compared with CDUS, with a kappa agreement of 0.23, a positive predictive value of 27%, and a negative predictive value of 93%. OST showed better performance in finger joints than in wrists (PIP AUC = 0.71 vs. wrist AUC = 0.58). The authors attributed this difference to the greater tissue thickness of the wrists, their proximity to large blood vessels, and the reduced space available for light transmission. Compared with clinical joint examination, OST did not improve sensitivity at comparable specificity. At 90% specificity, OST reached only 23% sensitivity. Nonetheless, the number of joints identified as inflamed by OST correlated significantly with established measures of disease activity, including VAS (*r* = 0.34), CRP (*r* = 0.25), and DAS28 (*r* = 0.31). Overall, OST demonstrated a statistically significant ability to detect joint inflammation compared with CDUS as the reference standard (AUC = 0.69, *p* < 0.001), with even higher performance observed in PIP joints specifically (AUC = 0.71) [31].

In 2015, Nair et al. assessed the cost-effectiveness of a simulated tight control treatment strategy using HandScan (TCHS) compared to usual care (UC) and a clinically assessed tight control strategy (TC) [15]. This evaluation included data from 299 patients with early RA and found that the TCHS strategy was highly cost-effective versus UC, saving an average of €4,660 per patient over two years. Compared with the TC strategy, TCHS resulted in similar costs and quality-adjusted life years (QALYs), with modest savings of €642 per patient on average. The authors concluded that incorporating HandScan could facilitate the implementation of tight control treatment strategies in clinical practice [15].

In a subsequent study published in 2016, van Onna et al. evaluated OST in 59 patients with RA, comparing its performance to US, as well as to clinical examination and MRI, using joint ultrasound scored according to the OMERACT methodology as the reference standard [49]. Diagnostic performance was assessed by ROC curve analysis. OST achieved good diagnostic accuracy for PIP (AUC = 0.79) and MCP joints (AUC = 0.78), but lower accuracy for wrist joints (AUC = 0.62). Across all joints, OST demonstrated an overall AUC of 0.81. The total OST score showed a moderate correlation with established measures of disease activity, with the highest correlation observed for US (*r* = 0.64) and the lowest for DAS28 (*r* = 0.42). OST also exhibited higher sensitivity for detecting synovitis than clinical examination: 83% versus 59% in PIP joints and 70% versus 42% in MCP joints, although specificity was lower (64% vs. 93% in PIP joints). Sensitivity for the wrist joints was similar between OST and clinical examination (39% vs. 37%). Notably, OST was able to detect inflammation even in patients in clinical remission or with low disease activity. The authors concluded that, in this pilot study, OST demonstrated moderate accuracy in detecting joint inflammation in RA and highlighted the need for further research to evaluate its responsiveness and validate its diagnostic value [49].

In 2018, Besselink et al. conducted a study involving 46 patients with rheumatoid arthritis (RA), using joint ultrasound (US) as the reference standard, with gray scale ultrasound (GSUS) and power Doppler ultrasound (PDUS) scored according to OMERACT criteria [17, 47, 48]. OST demonstrated a very good diagnostic performance compared to US, with AUC values of 0.83 for PIP joints, 0.88 for MCP joints, 0.74 for wrist joints, and 0.85 across all joints. In addition, the number of joints identified as inflamed by OST correlated strongly with US findings (*r* = 0.64). The study also demonstrated that certain pathological changes increased the risk of inflammation misclassification. In particular, dorsal erosions, osteophytes, and extensor tendon inflammation may contribute to false-positive or false-negative results. Overall, OST was considered a sensitive, specific, and objective method for assessing joint inflammation in the hands and wrists of RA patients. Despite the potential for misclassification in the presence of structural or tendon abnormalities, the authors concluded that OST is suitable for monitoring disease activity in RA patients without significant hand deformities [17].

In 2020, our study group assessed the diagnostic value of OST for identifying joint inflammation in patients with RA [35]. A total of 282 individuals participated in the study, which included 168 patients diagnosed with rheumatoid arthritis (RA) based on the ACR/EULAR classification criteria and 114 healthy individuals who served as the control group. Among the RA patients, a subgroup of 59 underwent joint ultrasound examinations (Power Doppler Ultrasound [PDUS] and Gray Scale Ultrasound [GSUS]). In this subgroup, OST showed moderate correlation with both PDUS (rho = 0.442, *p* = 0.001) and GSUS (rho = 0.591, *p* < 0.001). In the receiver operating characteristic (ROC) analysis of all joints (*n* = 1,251), OST displayed an area under the curve (AUC) of 0.67 (95% CI 0.631–0.709). The best diagnostic performance was observed at the wrist joints, with an AUC of 0.75 (95% CI 0.658–0.838), followed by the metacarpophalangeal (MCP) joints (AUC = 0.69, 95% CI 0.634–0.748) and the proximal interphalangeal (PIP) joints (AUC = 0.64, 95% CI 0.576–0.713). These findings suggest that OST measurements reflect both active inflammatory processes, as demonstrated by PDUS, and structural joint changes identified by GSUS [35]. In addition to exploring the relationships between OST and sonographic features, the study also specifically analyzed the associations with clinical and laboratory parameters. The OST values revealed significant, mostly moderate, correlations with established markers of disease activity. The strongest associations were found between OST and CRP (ρ = 0.389, *p* < 0.001), SJC (ρ = 0.361, *p* < 0.001), and DAS28-CRP (ρ = 0.364, *p* < 0.001). Further significant correlations were identified with DAS28-ESR, VAS, TJC, and ESR. All these correlations remained statistically significant even after adjusting for potential confounders such as age, sex, and BMI. Moreover, when disease activity was categorized according to DAS28 levels, patients in remission or with low disease activity (DAS28 < 2.6) had significantly lower OST values compared to those with moderate or high activity (*p* = 0.003). Overall, the findings indicate that OST values clearly correlate with established clinical and laboratory markers of disease activity [35].

In the following year, Verhoeven et al. published a letter commenting on the study by Triantafyllias et al. [50], focusing on the observed gender differences in OST scores. The authors confirmed that OST values were significantly higher in men than in women, both among patients with rheumatoid arthritis (RA) and in healthy controls, based on an analysis of 77 participants. They emphasized, however, that this sex-related discrepancy limits the comparability of disease activity between male and female RA patient groups when using the hand scan. Nevertheless, when serial OST measurements are used to monitor disease activity within individual patients, this gender difference becomes clinically irrelevant [50].

Triantafyllias et al. emphasized that a valid interpretation of OST values necessitates consideration of both disease-related and patient-related confounding factors, including sex, body mass index (BMI), and age, to enhance the diagnostic accuracy of this technology in rheumatoid arthritis (RA) and to better define its potential role in patient follow-up [51].

In a further OST/joint-US study, 61 RA patients were examined at a single time point to evaluate the effectiveness of HandScan in identifying remission as defined by joint US [32]. OST values of individual joints correlated significantly with ultrasound findings in both GSUS and PDUS for synovitis, degree of synovitis, and synovial thickness. Higher OST values were associated with an increased likelihood of synovitis in GSUS (OR 2.43) and with a higher degree of inflammation in GSUS (OR 3.80). However, these associations were significant only for MCP joints (*p* < 0.0001) and PIP joints (*p* < 0.044), but not for the wrists (*p* = 0.22), with the exception of synovitis grading. To identify US-defined remission, a cut-off value of 0.92 was determined for all 22 joints. This threshold achieved a sensitivity of 80.5%, a positive predictive value of 95.9%, and a specificity of 57.4%. Overall, the study demonstrated a possible value of OST in determining whether an individual joint is in remission on joint US. In addition, OST correlated significantly with ultrasound-detected signs of inflammation in both GSUS and PDUS [32].

**Fig. 5:**
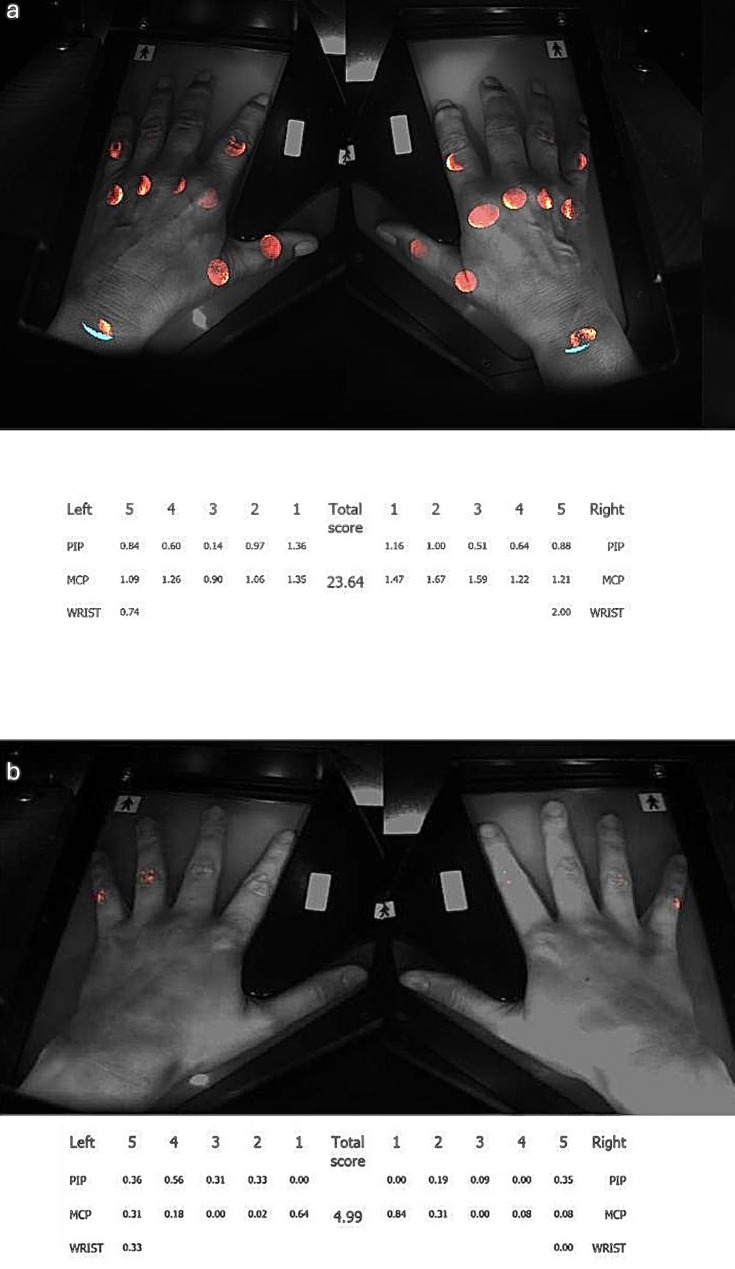
(**a**) PsA patient with OST values of 23.64; (**b**) Control subject with OST values of 4.99

The latest study, published in 2025, aimed to evaluate the diagnostic value of OST in patients with PsA [41]. OST values were compared to US and clinical disease activity markers in a study of 100 patients who met the CASPAR classification criteria [52] and 100 healthy controls. A total of 3000 joints per group were examined, including the MCP, PIP, DIP, and wrist joints, and a subgroup of 1020 joints was additionally examined using US. The PsA group had a significantly higher OST score (15.76) than the control group (10.24), (*p* < 0.001). Furthermore, the OST score showed good diagnostic performance in distinguishing between PsA and controls, with a sensitivity of 89% and a specificity of 71%. In addition, the OST score correlated significantly with established disease activity markers such as the PDUS (*r* = 0.412; *p* = 0.015), the GSUS (*r* = 0.419; *p* = 0.014), CRP (*r* = 0.232; *p* = 0.021), TJC (*r* = 0.203; *p* = 0.042), and VAS (*r* = 0.235; *p* = 0.019). The performance of OST compared to US resulted in an AUC of 0.678 (*p* < 0.001) for all joints, with the highest performance at the level of the wrist joints (AUC: 0.757), followed by the PIP joints (AUC: 0.648), the MCP joints (AUC: 0.636), and finally the DIP joints (AUC: 0.580). In addition, OST values were significantly higher in active joints (according to US) than in inactive joints (*p* < 0.001). In summary, OST was able to reliably distinguish between PsA patients and healthy controls and also correlated strongly with objective inflammation markers such as PDUS and GSUS [41]. An example of a measurement in a PsA patient can be seen in Fig. 5.

### Diagnostic performance of OST compared to MRI

MRI allows clear visualization of bone marrow edema as well as joint and soft tissue structures. However, it is an expensive procedure, not universally accessible, and is typically performed unilaterally [18, 53]. Several studies have directly compared OST with MRI results to assess correlations between these two diagnostic methods [34, 49].

The initial study addressing this issue was conducted by van Onna et al. in 2016. It involved 59 patients with rheumatoid arthritis (RA) and 10 control subjects experiencing non-inflammatory joint pain (arthralgia). The purpose of the study was to determine whether the OST could accurately measure disease activity in patients with RA [49]. MRI scans were conducted on the wrists and MCP joints of a single hand in patients with a DAS28 score of less than 2.6, mainly to identify subclinical signs of inflammation. The MRI images were assessed using the OMERACT Rheumatoid Arthritis MRI Score (RAMRIS) method. A significant correlation was observed between the OST value and the MRI synovitis score, with a correlation coefficient of *r* = 0.52 (*p* = 0.005) between OST and the RAMRIS Synovitis Score (RSS) in these patients who exhibited low or subclinical disease activity. The study highlighted that even individuals with low disease activity or clinical remission showed detectable subclinical inflammation on OST. However, no correlation was found between OST measurements and MRI-detected bone marrow edema, which the authors attributed to limited light penetration through bone tissue. Overall, MRI proved to be an essential reference standard for validating the OST technique, demonstrating that OST can detect circulation-related aspects of synovitis and confirming that subclinical inflammatory activity may persist even in patients who appear to be in remission [49].

**Fig. 6:**
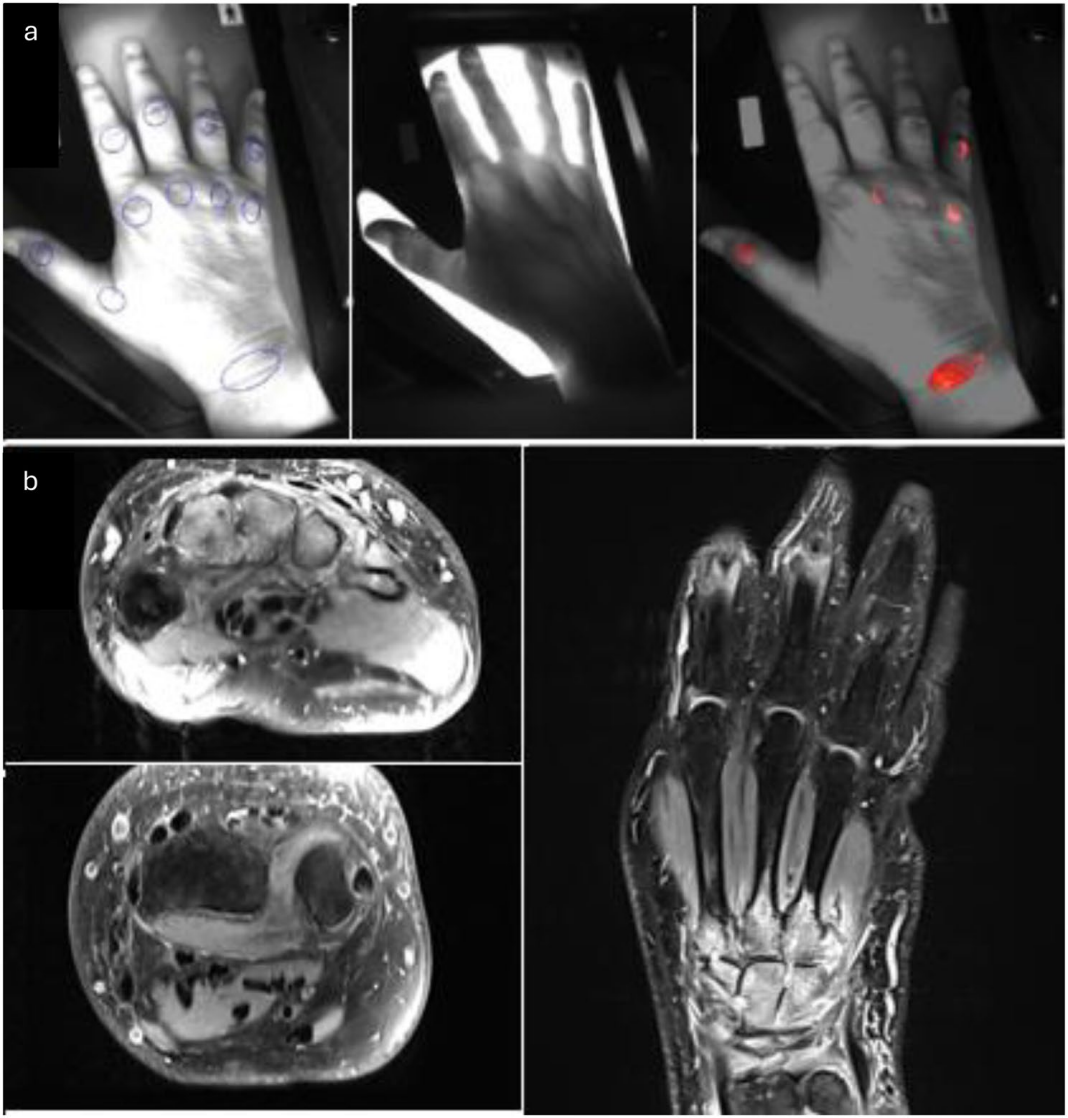
Comparison of OST and MRI in acute RA: (**a**) OST shows increased signal in wrist and MCP joints. (**b**) MRI indicates synovitis and bone marrow edema of the Hand

In 2025, our research group also investigated the performance of OST for assessing joint inflammation in comparison with MRI [34]. The study included 71 patients with IA, who were divided into two groups. The first group comprised patients with inflammatory signs on MRI (MRI+, defined as a RAMRIS synovitis score ≥ 1; *n* = 51), including individuals with RA, PsA, gout, and other forms of IA. The second group included patients without any synovitis on MRI (MRI-, *n* = 20). MRI+ patients had significantly higher OST scores (16.41 ± 5.53) compared with MRI- patients (11.52 ± 5.03) and healthy controls (10.78 ± 4.19), with all differences reaching high statistical significance (*p* < 0.001). Analysis of the entire hand revealed a significant correlation between OST values and the RAMRIS synovitis score (ρ = 0.541, *p* < 0.001). Significant correlations were also observed in separate assessments of the wrists (ρ = 0.644, *p* < 0.001), MCP joints (ρ = 0.582, *p* < 0.001), and PIP joints (ρ = 0.566, *p* < 0.001). In the MRI+ group, OST values showed a moderate correlation with the RAMRIS tenosynovitis score, particularly for MCP joints (ρ = 0.566, *p* < 0.001). Consistent with previous findings, no significant correlation was observed between OST values and the RAMRIS bone marrow edema score (ρ = 0.201, *p* = 0.106). The highest diagnostic performance of OST compared with the RAMRIS synovitis score was observed at the wrist joints, with an AUC of 0.833. Overall, the study concluded that OST reliably detects inflammatory activity relative to MRI, showing significant correlations with MRI-derived scores for both synovitis and tenosynovitis [34] (Fig. 6).

### Diagnostic performance of OST compared to clinical and laboratory parameters (cross-sectional studies)

Several studies have examined the correlations between OST and various clinical or laboratory parameters, comparing them with each other. The first study, conducted in 2012, included data from a total of 67 patients who had been diagnosed with rheumatoid arthritis (RA) according to the 2002 American College of Rheumatology (ACR) criteria [30]. The aim here was to test whether OST is suitable for the objective measurement of joint inflammation in RA patients. Only the values of the PIP joints with the highest inflammation were compared. As a reference method, a rheumatologist or experienced medical assistant assessed joint inflammation on a scale from 1 to 5, with 1 representing no inflammation and 5 severe inflammation. This was then statistically evaluated using PLS-DA. Good correlations were found between the OST measurements and the clinical assessment of joint inflammation, with an *r* = 0.63 (*p* < 0.001). The accuracy of the model was evaluated using a ROC curve and resulted in an AUC of 0.88. Furthermore, significant differences were found in the optical spectra of inflamed and non-inflamed joints (*p* < 0.001). During inflation of the arm cuff, more blood accumulated in the inflamed joints, where blood accumulation also occurred significantly faster (14.5 s) than in non-inflamed joints (21.6 s). In summary, OST measurements in this study correlated well with the clinical assessment of joint inflammation [30].

A study conducted by Wolkorte et al. in 2022 utilized an online survey targeting rheumatoid arthritis (RA) patients. The primary goal was to understand how these patients perceive their current disease status and to evaluate their assessment of the added value provided by the HandScan device [54]. Out of 408 respondents, 91% reported being satisfied with the number of doctor visits, which ranged from one to four times per year. Additionally, 73% felt they had a good understanding of their disease’s current status. However, only 59% expressed satisfaction with the frequency of imaging. Notably, a significant majority of respondents (95%) recognized the added value of using HandScan. This perceived benefit was particularly pronounced among those who had prior experience with HandScan (*p* = 0.04). Responders reported several reasons for this added value, including the device’s ability to measure the severity and presence of inflammatory activity in each joint individually [54].

A 2022 study by Verhoeven et al. analyzed data from 1,656 RA patients, who were divided into an internal (*n* = 1,505) and an external validation cohort (*n* = 151) [33]. The external cohort showed higher disease activity, with a mean DAS28 of 3.8. The study aimed to develop and validate a new disease activity index (DAS-OST) that replaced SJC and TJC with the OST score. Using DAS28-ESR as the reference standard, the final DAS-OST formula included ESR, PtGA VAS, gender, and the OST score. Agreement between DAS28-OST and DAS28 was excellent in the internal (*r* = 0.88; 95% CI: 0.87–0.90) and good in the external validation group (*r* = 0.82; 95% CI: 0.75–0.86). DAS-OST correctly classified patients with low disease activity (DAS28 < 3.2) with a sensitivity of 91%, and very high disease activity, with a specificity of 96%. In summary, DAS28-OST reliably estimates disease activity in RA [33].

In the same year, Verhoeven et al. published another study that evaluated the performance of the DAS-OST score compared to DAS28 for classifying rheumatoid arthritis as active or inactive [55]. In a cohort of 1,505 RA patients, the rheumatologist’s clinical classification of active versus inactive disease was used as the reference standard. The DAS28 demonstrated the highest discriminatory power (AUC-ROC 0.88; *p* < 0.01), while the DAS-OST performed significantly worse (AUC-ROC 0.78; *p* < 0.01). The OST score alone showed only low accuracy (AUC-ROC 0.59). Agreement with the clinical classification was higher for DAS28 than for DAS-OST. Moreover, DAS28 outperformed DAS-OST in identifying low disease activity, with a sensitivity of 87% and specificity of 72%, compared with 81% and 59%, respectively. Overall, the authors concluded that DAS-OST was statistically significantly less accurate than DAS28 for distinguishing active from inactive RA [55]. Although DAS-OST shows promise as a non-invasive and rapid method for assessing RA disease activity, its comparative accuracy versus DAS28 remains inconsistent across studies, suggesting that further research is required before widespread implementation.

A follow-up study by Hasan et al. included 484 participants, consisting of 313 women and 171 men [40]. This study examined the relationship between OST results and conventional assessments of RA disease activity. The total optical scores (TOS) of OST were significantly higher in patients with high DAS28 scores compared with those in remission, with low or moderate disease activity (*p* < 0.001). However, TOS could not reliably distinguish between remission, low, and moderate activity. TOS correlated significantly with DAS28-CRP (*p* = 0.006), 3-variable DAS (*p* = 0.009), SJC (*p* = 0.001), and TJC (*p* = 0.01), but not with ESR (*p* = 0.271) or CRP (*p* = 0.436). The OST score was also significantly higher in swollen than in non-swollen joints (*p* < 0.001), particularly in MCP2, MCP3, MCP4, and PIP1, but not in the wrists, MCP1, or PIP2. Overall, these findings suggest that while TOS lacks sufficient sensitivity to differentiate between remission, low, and moderate disease activity, OST shows promising utility for follow-up in established RA by accurately distinguishing swollen from non-swollen joints (β = 0.28, *p* < 0.001) [40].

In a small 2024 study conducted by Blanken et al., 24 RA patients and 37 controls were evaluated to assess the performance and influencing factors of OST [56]. Patients with RA showed higher mean OST scores (median 16.9) than controls (median 12.1). The OST score correlated moderately with ultrasound findings (β = 0.38; *p* = 0.001) and clinical disease activity (β = 0.30; *p* = 0.024; DAS28-CRP). In controls, higher OST scores were associated with BMI, hypertension, and male sex, but these factors were not significant in RA patients. The OST’s ability to detect inflammation, referenced to ultrasound and clinical swelling, showed an AUC of 0.63–0.70. Joint space narrowing and malalignment were linked to higher OST scores, whereas subchondral sclerosis was associated with lower values [56].

Triantafyllias et al. in our research group have also evaluated OST in relation to clinical activity parameters in several studies [35, 36]. In 2024, we published a study focused on developing patient-specific cut-off values for the OST to improve diagnostic accuracy. The study included 225 participants: 72 with RA, 53 with non-inflammatory arthralgia (NIA), and 100 healthy controls. The OST demonstrated strong diagnostic performance in distinguishing RA from healthy controls (AUC = 0.810), improving further in patients with at least one swollen joint (AUC = 0.841). When comparing RA and NIA, performance remained high (AUC = 0.822). Gender-specific cut-off values were defined for active disease: 11.2 for women (sensitivity 93%, specificity 71.2%) and 18.2 for men (sensitivity 67%, specificity 90%). OST correlated significantly with SJC (ρ = 0.355, *p* = 0.002), DAS28-ESR (ρ = 0.361, *p* = 0.002), and VAS (ρ = 0.383, *p* = 0.001), but not with CRP, ESR, or TJC. Predictive models incorporating OST, age, and gender achieved optimal accuracy for estimating RA probability. Overall, the study demonstrated that gender-specific thresholds and predictive models can enhance the diagnostic accuracy of hand scans [36].

## OST in longitudinal studies

In 2021, Verhoeven et al. conducted an observational cohort study to investigate the ability of HandScan to measure disease activity in patients with rheumatoid arthritis over time [57].

A total of 64 patients with RA participated in the study, which included 32 individuals with early RA who were starting disease-modifying antirheumatic drugs (DMARDs) and 32 individuals with established RA who were either initiating or switching to TNF blockers. Patients were assessed at four different time points: baseline, and after 1, 3, and 6 months. Both the OST and the Disease Activity Score 28 (DAS28) were recorded. The study aimed to explore the longitudinal associations between OST and DAS28, as well as to evaluate whether short-term changes in OST could predict therapeutic response. The results showed that OST scores were significantly associated with DAS28. However, incorporating OST into a predictive model only increased the explained variance from 57% to 59%, indicating that its contribution is limited. The correlations observed between OST and DAS28 at each time point ranged from weak to moderate (ρ = 0.18–0.39). Importantly, short-term changes in OST or baseline OST did not significantly predict clinical response (as measured by the ACR or EULAR criteria) at 3 or 6 months. In contrast, short-term changes in DAS28 proved to be strong predictors [57]. Although OST showed only a weak correlation with DAS28 and did not predict short-term response, it consistently reflected disease activity and may serve as a useful adjunct to traditional RA assessments.

Another study from 2021 by van Boheemen et al. investigated whether OST is useful in individuals at risk for RA to detect subclinical inflammation and predict the subsequent development of arthritis [58]. In this prospective study, individuals at risk for RA (*n* = 35), RA patients (*n* = 24), and healthy controls (*n* = 37) were examined using OST, with individuals at risk additionally undergoing an ultrasound examination. The development of arthritis was tracked over a median of 27 (range 26–28) months. No significant correlation was observed between OST and ultrasound in at-risk individuals (ρ = 0.01), and OST could not predict the development of arthritis. OST scores in the at-risk group did not differ from healthy controls (*p* = 1.000), whereas RA patients had significantly higher scores than both controls and at-risk individuals. Overall, this study indicates that hand scanning is currently not useful for predicting or diagnosing arthritis in individuals at risk for RA [58].

In 2023, our study group investigated whether OST can reliably assess the response to glucocorticoid pulse therapy in patients experiencing an active arthritis flare [14]. The observational study included 39 RA patients, 11 with psoriatic arthritis, 4 with gout, and 114 healthy controls. Patients had high disease activity prior to glucocorticoid (median baseline DAS28: 5,12). OST, ultrasound (GSUS and PDUS), and clinical and laboratory parameters were measured before and median 4 days after glucocorticoid therapy. Using a Bayesian approach, changes in OST (ΔOST) and PDUS (ΔPDUS) did not differ significantly for MCP (accuracy 68.1%), PIP (60.4%), or wrist joints (50.4%), indicating similar diagnostic potential. ΔOST and ΔGSUS were also comparable for MCP (51.1%) and PIP joints (78.7%), though differences were significant for the wrist. OST distinguished healthy controls from arthritis patients both before (AUC 0.882) and after therapy (AUC 0.81), and correlated moderately with SJC (ρ = 0.417, *p* = 0.002), but not with TJC, ESR, or total DAS28. Overall, OST appears comparable to ultrasound in assessing therapy response ([14].

In a 2025 study, our group examined the long-term correlations between OST values and clinical disease activity markers to assess the ability of the hand scan to monitor disease progression [39]. RA (*n* = 40), PsA-SpA (*n* = 15), and gout patients (*n* = 5), along with 114 healthy controls, were assessed at two time points in a retrospective, longitudinal study. Changes in OST (ΔOST) were compared with changes in clinical and laboratory parameters (e.g., ΔDAS28). Patients had significantly higher mean OST values than controls at both time points (time point a: 15.89; time point b: 15.40 vs. controls: 10.79; *p* < 0.001). OST correlated significantly with DAS28-ESR, DAS28-CRP, SJC, and TJC at both time points, but not with CRP or ESR. Importantly, ΔOST correlated with changes in clinical scores: ΔOST vs. ΔDAS28-ESR (*r* = 0.377, *p* = 0.004), ΔOST vs. ΔDAS28-CRP (*r* = 0.355, *p* = 0.007), and ΔOST vs. ΔSJC (*r* = 0.488, *p* < 0.001). Overall, OST demonstrates good diagnostic performance and significant long-term correlations with established clinical activity markers [39].

The latest long-term study conducted in 2025 included 112 patients who were newly diagnosed with rheumatoid arthritis (RA) and had not previously received disease-modifying anti-rheumatic drugs (DMARDs) [59]. It aimed to determine whether a treat-to-target (T2T) strategy based on HandScan remission is non-inferior to one based on ACR/EULAR 2011 Boolean remission. All patients initially received MTX 10 mg/week and prednisone 10 mg/day, with escalation to MTX 30 mg, prednisone 10 mg, and a TNF inhibitor, if remission was not achieved. HandScan remission was defined as ≤ 1 joint with OST > 1 and total OST ≤ 11, while Boolean remission followed standard ACR/EULAR criteria [59]. After 18 months, disability measured by HAQ increased 0.21 units in the HandScan group compared with the Boolean group (95% CI 0.01–0.40, *p* = 0.04), exceeding the pre-specified non-inferiority margin of 0.2, so non-inferiority could not be established [52]. Non-adherence to treatment was high in both groups, with rates of 95% for HandScan and 80% for Boolean. This was mainly attributed to perceived overtreatment, and a higher treatment intensity significantly increased the risk of non-adherence (HR 1.3, 95% CI 1.1–1.5, *p* = 0.02). Some patients who discontinued therapy were already in remission, as indicated by a mean DAS28 score of 2.0. The remission rates were highest among patients in this DAS28 category but lowest in the HandScan group. Overall, the remission criteria set by HandScan appear to be too strict for clinical practice, which could result in over-treatment and poorer outcomes. Additionally, non-adherence is still a concern even with the standard Boolean targets [59].

**Table 1:** List of included studies

	Study	Year of publication	Objective	Diagnosis	Number of included patients	Control	Reference method	AUC-ROC	Main results
1	Meier et al.	2012	Evaluation of the potential of optical attenuation measurements for determining degrees of joint inflammation	RA	79	No	Clinical assessment of joint inflammation (1 to 5 point scale)	0.88 (for the joint with the highest inflammation)	The correlation between optical measurement and clinical assessment of PIP jointinflammation was r = 0.63 (p < 0.001). Diagnostic accuracy showed an AUC of 0.88
2	Nair et al.	2015	Evaluation of the cost-effectiveness of a tight control treatment strategy using the HandScan (TCHS) compared to purely clinical evaluation (TC) and usual care (UC) in early RA	RA	299	Yes Usual care (UC, 148 subjects) and Tight control (TX, 151 subjects)	Economic evaluation based on a Markov model that extrapolated QALYs and costs over 2 years. Clinical data (DAS28) were used from the CAMERA study		Compared to UC, TCHS resultedin cost savings (€4,660 saved) and a QALY gain (0.06 QALYs gained). TCHS and TC resulted in comparable costs and QALYs. A tight control strategy (TC/TCHS) is highly cost- effective in early RA compared to a non-tight control approach (UC)
3	Van Onna et al.	2016	Testing whether OST can validly measure disease activity in RA patients	RA	59	Yes 10 controls with non- inflammatory arthralgia.	US (Grey- Scale/PowerDoppler), MRI (RAMRIS)	Total: 0.81 (all joints), PIP: 0.79,MCP: 0.78, wrist:0.62	Performance (vs. US) was good atPIP (AUC 0.79) and MCP (AUC 0.78, both p < 0.0001) but poorer at the wrist (AUC *0.62). OST correlated moderately with DAS28 (r = 0.42, p = 0.001) and more strongly with US scores (r = 0.64, p < 0.0001)
4	Krabbe et al.	2016	Investigation of thesensitivity and specificity of OST in detecting joint inflammation in RA	RA	62	No	Color DopplerUltrasonography (CDUS)	Total: 0.69 (all joints), PIP: 0.71,MCP: 0.60, wrist:0.58 (not significant)	OST correlated with VAS(r=0.34), CRP (r=0.25), DAS28(r=0.31), and HAQ (r=0.35). The Kappa agreement value between OST and colour Doppler ultrasound (CDUS) for all joints examined was 0.23. OST measurements showed a sensitivity of 54% and a specificity of 79% for all joints combined
5	Besselink et al.	2018	Development of an OST model for measuring joint inflammation and evaluating (patho-)physiological findings that could lead to misclassifications	RA	46	No	US-Scores (GSUS and PDUS)	Total: 0.85 (all joints), PIP: 0.83, MCP: 0.88, wrist: 0.74	OST showed very good diagnostic performance compared to US (AUC-ROC 0.85 across all joints). At thepatient level, OST correlated moderately (r= 0.54) with US, at the jointlevel, US correlation with OST was strong (r= 0.64)
6	Triantafyllias et al.	2020	Validation of previous OST study results, investigation of OST differences between RA patients and controls, and investigation of the relationships between OST and patient- and disease- related characteristics	RA	168	Yes 114 healthy controls	Clinical/laboratory markers (DAS28- ESR/CRP, SJC, TJC, CRP, ESR); US (GSUS, PDUS) in subgroup (n=59)	RA vs. healthy controls: 0.71 (total score), RA subgroup vs. controls: 0.76, PIP: 0.64, MCP: 0.69, wrist: 0.75	RA patients showed higher OST values (14,55) than controls (10,32, p < 0.001). OST correlated moderately with CRP (rho 0.389, p < 0.001), SJC (rho 0.361, p < 0.001), DAS28-ESR (rho 0.313, p < 0.001), and DAS28-CRP (rho 0.364, p < 0.0019).In the US subgroup, OST correlated moderately with GSUS (ρ 0.591, p < 0.001) and with PDUS (ρ 0.442, p = 0.001)
7	Van Boheemen et al.	2021	Comparison of OST with ultrasound for detecting subclinical inflammation in at-risk individuals and correlation with subsequent onset of arthritis	Patients with RF and/or anti- citrullinated protein antibody (ACPA)- positive arthralgia without clinical arthritis	35	Yes 24 RA patients and 37 healthy controls	US (GSUS, PDUS)		In at-risk individuals, OST did not correlate with US. OST scores did not differ significantly between at- risk individuals and healthy controls (P = 1.000). Of four individuals who developed arthritis, only one had an OST score above the cut-off, while three showed GS signal and two showed PD signal. It is unlikely that OST can contribute to the diagnosis and prediction of arthritis in individuals at risk for RA
8	Verhoeven et al.	2021	Investigation and quantification of gender differences and other potential confounding factors (age, BMI, palm size/volume) in OST scores in RA patients and controls	RA	77	Yes 37 controls	DAS28, gender, and anthropometric data		OST scores tended to be higher in men than in women in both RA patients and controls, suggesting a gender difference. The difference was significant in controls (P = 0.05). In male participants, age (negative) and palm size (positive) were predictors of OST score; in female participants, BMI (negative) and hand volume (positive) were predictors
9	Verhoeven et al.	2021	Determining the ability of the HandScan (OST) to measure RA disease activity longitudinally and whether changes in OST scores predict treatment efficacy	RA	64	No	DAS28		Longitudinal association between the OST score and DAS28: A one- unit change in the OST score was associated with an average of 0.03 change in DAS28 (95% CI: 0.01, 0.06, P = 0.03). The baseline OSTscore was not a predictor of a good EULAR response
10	Wolkorte et al.	2022	Investigation of the patient perspective regarding their insight into their current disease status and the potential added value of HandScan for inflammation monitoring	RA	408	No	patient perspective		73% of respondents felt they had sufficient insight into their disease status. 95% saw little or significant added value in HandScan. The main reason for the added value was the provision of clear images of inflammatory activity and its location. Most patients (47%) preferred a HandScan examination at every hospital visit
11	Hasan et al.	2022	Investigation of the association between the TOS and DAS28, and between OST and joint swelling at the joint level	RA	484	No	DAS28		TOS correlated with DAS28 (B=0.36, p<0.001). OST was significantly higher in swollen joints than in non-swollen joints (B 0.28, P < 0.001). TOS only significantly differentiated high disease activity from other conditions
12	Verhoeven et al.	2022	Development and validation of a composite RA disease activity index (DAS-OST) using OST scores as a substitute for painful and swollen joint counts	RA	1,505 (development/ internal validation) + 151 (external validation) observations	No	DAS28-ESR	DAS-OST (including PtGA VAS, internal validation): Remission: 0.93; LDA: 0.92; HDA: 0.97; DAS-OST (including PtGA VAS, external validation): Remission: 0.95; LDA: 0.93; HDA: 0.92	DAS-OST could be accurately estimated using OST score, ESR, PtGA VAS, and gender. ICC between DAS-OST and DAS28: 0.88 (internal), 0.82 (external)
13	Verhoeven et al.	2022	To determine the value of a modified DAS (DAS-OST) – without joint counts but with a HandScan score (OST) – in comparison to DAS28 for classifying rheumatoid arthritis (RA) as active versus inactive	RA	1505	No	DAS28	highest AU-ROC of 0.88 (95%CI 0.85- 0.90) was shown for DAS28; AU- ROC of DAS- OST: 0.78	The highest AU-ROC value for DAS28 was 0.88 (95% CI 0.85– 0.90), while the AU-ROC value for DAS-OST was 0.78 (95% CI 0.75–0.81). Compared to DAS28, DAS-OST classified RA as statistically significantly worse than active versus inactive (difference 0.10, p<0.01). It is suggested that a DAS modification without joint scores could be useful in strategies to limit routine outpatient visits to rheumatologists
14	Triantafyllias et al.	2023	Investigation of the performance of OST in assessing response to GC therapy in active arthritis and investigation of associations with clinical, laboratory, and US activity markers	Arthritis (RA, PsA, peripheral SpA, gout)	54	Yes114 individuals without underlying inflammatory diseases, arthralgia, or clinical signs of osteoarthritis	US (GSUS, PDUS)		OST was able to evaluate the response to therapy in a similar way to joint US. The distributions of ΔPDUS and ΔOST did not differ significantly (Accuracy MCP: 68.1%; PIP: 60.4%; Wrists: 50.4%)
15	Blanken et al.	2024	Investigation of the performance and influencing factors of OST as a new technique for measuring joint inflammation in RA	RA	24	Yes 37 controls	Ultrasound (US), clinical disease parameters (DAS28- CRP), radiological features		The mean OST score was higher in RA patients than in controls (16.9 vs. 12.11). At the patient level, the OST score was moderately associated with US (beta 0.38 (95% CI 0.16–0.60), p = 0.00) and clinical disease activity (DAS28-CRP) (beta 0.30(95% CI 0.04– 0.57), p = 0.024). In controls, male sex, high BMI, and hypertension were associated with higher OST scores, but these associations were absent in RA patients
16	Rinkin et al.	2024	Investigation of the effectiveness of HandScan (OST) for identifying US- defined RA remission	RA	61	No	US (GSUS, PDUS)		HandScan can identify US-defined remission in RA. OST correlated significantly with GSUS (OR 2,43, p < 0,0001) and PDUS parameters (OR 3,72, p = 0,0002)
17	Triantafyllias et al.	2024	Proposal of patient-adjusted OST cut-off values and development of clinical models to predict the probability of early RA diagnosis	RA	72	Yes100 healthy controls; 53 patients with non- inflammatory arthralgia	Clinical and laboratory evaluation	RA vs. healthy controls: 0.810, active RA (≥1 swollen joint) vs. controls: 0.841, RA vs. non- inflammatory arthralgia: 0.788	Gender-specific cut-offs for active disease: 11.2 (women, sensitivity 93%, specificity 71.2%) and 18.21 (men, sensitivity 67%, specificity 90%).OST correlated moderately/significantly with DAS28 (r = 0.42, p < 0.001), SJC (ρ = 0.355, p = 0.002) and VAS (ρ = 0.383, p = 0.001)
18	Triantafyllias et al.	2025	Investigation of longitudinal correlations between OST and clinical disease activity markers (DAS28/SJC/TJC) under real-world conditions	Arthritis (RA, PsA, peripheral SpA, gout)	60	Yes114 healthy controls	Clinical activity parameters (DAS28, SJC)		ΔOST values were significantly associated with ΔDAS28 (r=0.377) and ΔSJC (r=0.488). OST showed good diagnostic performance (AUC 0.781 vs. controls)
19	Triantafyllias et al.	2025	Investigation of the performance of OST in assessing joint inflammation compared to MRI in patients with inflammatory arthritis (IA)	Inflammatory arthritis (IA) (RA, PsA, gout, MCTD, undifferentiated arthritis)	71	Yes 114 healthy controls	MRI, Clinical activity parameters (DAS28, SJC)	IA (MRI+) vs. healthy controls: 0.800, wrist (vs. RAMRIS synovitis): 0.833, OST vs. DAS28 (IA+): 0.670	OST showed similar performance to MRI. Significant correlations between OST and RAMRIS synovitis (rho = 0.541, p < 0,001) and RAMRIS tenosynovitis (rho = 0.341, p = 0,010). Best diagnostic performance at the wrist level (AUC 0.833)
20	Triantafyllias et al.	2025	Evaluation of the value of OST in detecting joint inflammation in PsA patients and assessment of correlations with US and clinical activity markers	PsA	100	Yes100 healthy controls	US (PDUS, GSUS)	PsA vs. healthy controls: 0.848, PIP: 0.648, MCP: 0.636, wrist: 0.757, DIP: 0.580	OST was significantly higher in PsA patients than in controls (15.76 vs. 10.24; p<0.001). Good diagnostic performance (AUC 0.848). Significant correlations with PDUS (ρ 0,412, P = 0,015), DAPSA and DAS28
21	Van der Leeuw et al.	2025	Investigation of the non- inferiority of HandScan- guided treatment compared to clinically DAS-guided treatment in early RA	RA	112	No	ACR/EULAR 2011 Boolean Remission		The current HandScan remission criteria are unsuitable for a T2T strategy in early RA, as they can lead to overtreatment and poorer clinical outcomes (based on HAQ score)

## Discussion

This SLR provides the first comprehensive synthesis of published data on OST as a tool for assessing joint inflammation in patients with RA and other inflammatory arthritides. In both cross-sectional and longitudinal studies, OST demonstrated consistent correlations with established imaging modalities, including joint-US and MRI. It also showed moderate associations with clinical and laboratory markers of disease activity such as DAS28, SJC, CRP, and ESR. The diagnostic accuracy of OST varied by joint region, being typically highest in the wrists, followed by the MCP and PIP joints. This accuracy was influenced by study design and disease activity. Importantly, OST also showed the ability to detect subclinical inflammation and to identify joints that were in remission, as defined by other well-established imaging methods.

Overall, OST provides several beneficial features that make it an effective tool for assessing patients with inflammatory arthritis. Notably, OST is non-invasive and quick, allowing for the simultaneous evaluation of both hands and wrists in about 1.5 min, all without causing pain or requiring contrast agents [39, 49]. Its automated software analysis minimizes examiner bias, making measurements independent of operators and enabling trained non-specialist personnel to perform assessments reliably [49]. Additionally, OST offers inflammation quantification through individual joint scores and a total optical score, providing reproducible metrics for disease activity [14, 34, 57]. OST can be sensitive to subclinical inflammation, detecting synovitis even in patients with low disease activity [49, 57]. However, its diagnostic performance tends to decline at lower levels of disease activity [36]. Longitudinal studies indicate that OST effectively monitors therapeutic responses, with changes closely reflecting anti-inflammatory treatment and showing outcomes similar to power Doppler and grayscale ultrasound in most joints [14].

However, several disadvantages of OST should be considered. Changes in structural joints, such as bone erosions and osteophytes, can affect OST measurements, and therefore, limitations in accuracy must be acknowledged [56]. The technique does not detect bone marrow edema, which limits its comparability with MRI [34]. Diagnostic performance can vary across joint categories due to anatomical differences, tissue thickness, proximity to major vessels, and variable light penetration. Moreover, the lack of widely validated cut-off scores further complicates interpretation in clinical practice. OST values can be influenced by patient-specific characteristics such as gender, hand size, and in some cases age, emphasizing the need for patient-adapted cut-off thresholds to ensure accurate assessment [33, 35, 36]. Additionally, the HandScan device has limited joint coverage, as it assesses disease activity only in the finger and wrist joints, whereas other joints commonly affected in RA, such as the shoulders, elbows, or knees, are not evaluated. Collectively, these factors may restrict the utility of OST as a standalone tool in routine clinical practice, particularly for patients with extensive or atypical joint involvement.

When discussing the existing studies, it is important to note several strengths and limitations. One of the main strengths is that most studies used validated reference standards, such as OMERACT-scored ultrasound or RAMRIS MRI. This approach ensures that comparisons involving OST against these well-established imaging methods are reliable and clinically significant [14, 17, 31, 32, 34, 41, 49].

In addition, several studies compared OST with clinical and laboratory parameters, offering a more comprehensive assessment of its performance, including in settings without access to advanced imaging and in relation to traditional disease activity markers [33, 36, 40]. The inclusion of both cross-sectional and longitudinal study designs allowed for the evaluation of diagnostic accuracy at a single time point, as well as tracking changes in disease activity over time and monitoring treatment response [14, 39]. Some studies have limitations due to being small, single-center, or pilot investigations, which may affect their generalizability. Additionally, differences in joint selection, scoring methods, and the lack of standardized cut-off definitions make direct comparisons challenging. There is also limited data on non-rheumatoid arthritis inflammatory arthritides, such as psoriatic arthritis, which raises questions about the broader applicability of OST in various patient populations [41]. Moreover, some studies did not account for potential confounding factors, such as patient-specific characteristics including gender, hand size, body mass index, or age, which, may influence OST results and may affect the interpretation of findings in routine clinical practice.

The future prospects of OST highlight several unmet needs. Current findings are largely limited to small peripheral joints, underscoring the need for studies to evaluate applicability to larger joints such as the elbow, shoulder, ankle, and knee. The application of OST in the axial skeleton, particularly at the sacroiliac joints, remains unexplored, likely due to limited light transmission and anatomical constraints. Furthermore, the lack of a validated scoring system limits comparability between studies and clinical implementation. Future research should also explore artificial intelligence-based approaches for improved signal interpretation, prediction of treatment response, and detection of early inflammatory or erosive changes.

## Conclusion

OST has emerged as a promising, non-invasive, and rapid imaging modality for assessing joint inflammation in inflammatory arthritis. By integrating OST data with established clinical measures and patient-reported outcomes, this technique has the potential to enhance the objectivity and efficiency of disease activity monitoring. In particular, OST may complement ultrasound, MRI, and clinical joint assessment as a practical tool in routine care. However, further multicenter studies and clinical trials are needed to standardize measurement protocols, refine scoring systems, and validate its performance across diverse patient populations.

## Supplementary Information

Below is the link to the electronic supplementary material.

Supplementary Material 1

## Data Availability

All data were obtained from previously published studies.

## Abbreviations

ACR: American College of Rheumatology
AUC: Area Under the Curve
AUC-ROC: Area Under the Receiver Operating Characteristic Curve
BMI: Body Mass Index
CASPAR: Classification Criteria for Psoriatic Arthritis
CDUS: Color Doppler Ultrasonography
CRP: C-reactive Protein
DAPSA: Disease Activity in Psoriatic Arthritis
DAS28: Disease Activity Score in 28 joints
DIP: Distal Interphalangeal joints
DMARDs: Disease-Modifying Antirheumatic Drugs
ESR: Erythrocyte Sedimentation Rate
EULAR: European Alliance of Associations for Rheumatology
FOI: Fluorescence Optical Imaging
GSUS: Gray-scale Ultrasound
HAQ: Health Assessment Questionnaire
IA: Inflammatory Arthritis
MCP: Metacarpophalangeal joints
MRI: Magnetic Resonance Imaging
MTX: Methotrexate
OA: Osteoarthritis
OMERACT: Outcome Measures in Rheumatoid Arthritis Clinical Trials
OST: Optical Spectral Transmission
PDUS: Power Doppler Ultrasound
PIP: Proximal Interphalangeal joints
PLS-DA: Partial Least Squares Discriminant Analysis
PRISMA: Preferred Reporting Items for Systematic Reviews and Meta-Analyses
PsA: Psoriatic Arthritis
PtGA: Patient Global Assessment
QALY: Quality-Adjusted Life Year
RA: Rheumatoid Arthritis
RAMRIS: Rheumatoid Arthritis MRI Score
ROC: Receiver Operating Characteristic
ROI: Region(s) of Interest
RSS: RAMRIS Synovitis Score
SLR: Systematic Literature Review
SJC: Swollen Joint Count
SpA: Spondyloarthritis
T2T: Treat-to-Target
TC: Tight Control
TCHS: Tight-Control strategy using HandScan
TJC: Tender Joint Count
TOS: Total Optical Score
UC: Usual Care
US: Ultrasound
VAS: Visual Analogue Scale
