# Supplementary material for: Assessing disease activity in inflammatory arthritis using optical spectral transmission: a systematic review compared to joint ultrasound, MRI, and clinical activity markers
Source: BMC Rheumatol. 2026 Mar 18;10:32. doi: 10.1186/s41927-026-00635-x (PMC13063693; doi:10.1186/s41927-026-00635-x)
Supplement: Supplementary file 1 — Supplementary Material 1 [file 41927_2026_635_MOESM1_ESM.docx]

**Data on osteoarthritis**

In 2019, Besselink et al. conducted a study comparing OST with ultrasound (US) in 47 osteoarthritis (OA) patients, 89% of whom were female (38). This cross-sectional study aimed to evaluate the suitability of OST for detecting synovitis in the hands and wrists of OA patients, using US as the reference standard. Synovitis was assessed according to standardized OMERACT guidelines, using both power Doppler (PDUS) and gray-scale US (GSUS) scored from 0 to 3. OST measurements were performed before and after US assessment. Associations between OST and US-detected synovitis, expressed in standard deviation (SD) units, were Δ0.37 SD for all joints combined (p < 0.001), Δ0.81 SD for PIP joints (p < 0.001), Δ0.14 SD for DIP joints (p = 0.484), and Δ0.37 SD for wrist joints (p = 0.178), independent of dorsal vascularity and osteophytes. The diagnostic performance of OST, assessed using the area under the receiver operating characteristic curve (AUC-ROC), was 0.74 for all joints combined (p < 0.001), 0.69 for PIP joints (p < 0.001), 0.61 for the wrists (p = 0.234), and 0.54 for DIP joints (p = 0.486). Overall, OST scores were significantly associated with synovitis, particularly in the PIP joints, and this association was independent of other OA characteristics such as dorsal vascularity or osteophytes. Performance was, however, poor for DIP and wrist joints (38).

38. Besselink NJ, Jacobs JWG, Westgeest AAA, van der Meijde P, Welsing PMJ, Marijnissen ACA, et al. Can optical spectral transmission assess ultrasound-detected synovitis in hand osteoarthritis? PLoS One. 2019;14(2):e0209761. Epub 20190222. doi: 10.1371/journal.pone.0209761. PubMed PMID: 30794572; PubMed Central PMCID: PMC6386475.
